# *Notes from the Field:* Gastrointestinal Illness Among Hikers on the Pacific Crest Trail — Washington, August–October 2022

**DOI:** 10.15585/mmwr.mm7236a5

**Published:** 2023-09-08

**Authors:** Arran Hamlet, Katherine Begley, Shanna Miko, Laurie Stewart, Waimon Tellier, Joenice Gonzalez-De Leon, Hillary Booth, Soyeon Lippman, Amy Kahler, Alexis Roundtree, April Hatada, Scott Lindquist, Beth Melius, Marcia Goldoft, Mia Mattioli, Michelle Holshue

**Affiliations:** ^1^Epidemic Intelligence Service, CDC; ^2^Washington State Department of Health; ^3^Laboratory Leadership Service, CDC; ^4^Division of Foodborne, Waterborne and Environmental Diseases, CDC; ^5^Viral and Rickettsial Disease Laboratory, California Department of Public Health; ^6^Career Epidemiology Field Officer Program, CDC.

On August 26, 2022, the Washington State Department of Health received informal reports of numerous Pacific Crest Trail hikers with acute gastroenteritis (AGE). The Pacific Crest Trail stretches 2,650 miles from California to Washington, attracting hikers from around the world (*1*). An investigation of social media postings on September 5 found 27 reports of AGE by Washington Pacific Crest Trail hikers during the previous month, 26 of whom provided information about symptom onset date (Figure). Numerous additional reports without a specific date were found, suggesting that that AGE was occurring during the 2022 hiking season.

**FIGURE:**
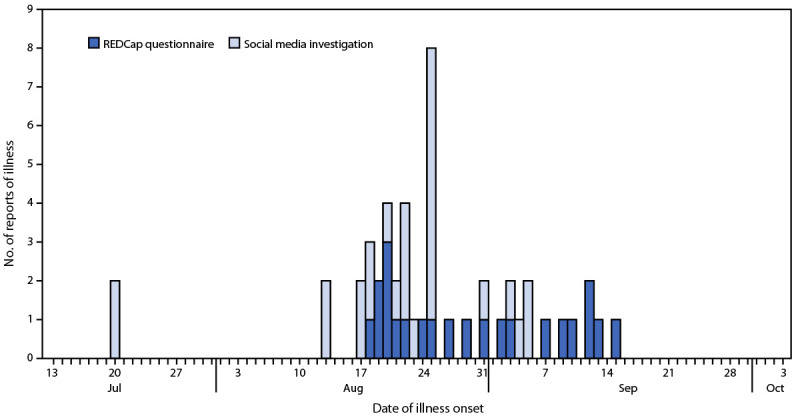
Number of hikers with gastrointestinal symptoms by illness onset date* based on social media investigation (N = 26) and REDCap Survey (N = 22) — Pacific Crest Trail, Washington, July 20–October 4, 2022 * Among a total of 27 social media postings and 27 REDCap survey responses, 26 and 22 persons, respectively, reported the date of symptom onset and are included in this figure.

## Investigation and Outcomes

During September 13–18, a REDCap survey was posted on a Facebook group popular with Washington Pacific Crest Trail hikers and displayed (with a quick response code) at trailhead locations where illness had been reported. Survey responses were collected from 27 ill Pacific Crest Trail hikers regarding symptoms, locations, and contact details; 22 respondents reported onset dates. Two respondents (the only two with symptoms during the preceding 14 days who were still in Washington), agreed to provide stool samples; both samples tested positive for norovirus by real-time reverse transcription–polymerase chain reaction (RT-PCR) (*2*) at the Washington State Public Health Laboratory. The samples were sent to CaliciNET Laboratory (California Department of Public Health) for sequencing; both were identified as GII.10[P16]. Twenty (74%) survey respondents reported an illness of short duration (median = 2.5 days; 95% CI = 1–15.7 days); among 22 (81%) who reported signs and symptoms, those most commonly reported were fatigue (21; 95%) and vomiting and diarrhea (17; 77%). Twenty-one (95%) survey respondents who reported an onset date noted that they became ill within a 73-mile stretch of the Washington Pacific Crest Trail; this suggested the potential for environmental exposure. 

The probable distance an infected hiker could have walked during the norovirus incubation period was subtracted from onset locations to estimate a probable geographic exposure area. Cross-referencing the estimated exposure area with facilities on the trail guided identification of two sets of ventilated improved pit (VIP) latrines, a cabin available as a rest stop, and a stream with drinking water as potential sampling sites. During October 3–4, 2022, samples were collected from drinking water sources used by hikers and high-touch surfaces in the cabin and VIP latrines to test for norovirus and fecal contamination using real time RT-PCR and quantitative PCR testing (*3*,*4*). Norovirus was not detected in any samples. No culture-based fecal indicators, *E. coli*, or human-specific fecal contamination were detected in any water source. All surface swabs inside the cabin and pit latrines tested positive for human-specific fecal contamination. Despite absence of detection of norovirus from environmental sampling, symptom profiles, respondent and environmental laboratory results, and epidemiologic links all supported the conclusion that the outbreak was primarily caused by norovirus, and that exposure to contaminated surfaces within the cabin and VIP latrines likely amplified transmission. Improved sanitation protocols, messaging on handwashing, and guidance on reporting outbreaks were shared with jurisdictional authorities. This activity was reviewed by CDC and was conducted consistent with applicable federal law and CDC policy.*

## Preliminary Conclusions and Actions

Although the REDCap survey identified only 27 ill hikers, social media reports indicated that the true size of the outbreak was likely substantially larger, with 27 reports with a date of onset, and numerous others without further chronologic information apart from the year (2022). Norovirus prevention in remote areas is difficult because of a lack of easily available clean water and soap for handwashing, and inability to routinely disinfect shared surfaces (e.g., cabins and restrooms). Moreover, alcohol-based hand sanitizers, commonly used in hiking, are not effective against norovirus (*5*). Preventing future outbreaks will require fostering increased awareness of the importance of handwashing and lack of effectiveness of alcohol-based hand sanitizers against norovirus, and more frequent cleaning of public facilities; early outbreak detection might be facilitated by social media surveillance.

## Footnotes

* 45 C.F.R. part 46.102(l)(2), 21 C.F.R. part 56; 42 U.S.C. Sect. 241(d); 5 U.S.C. Sect. 552a; 44 U.S.C. Sect.3501 et seq.
